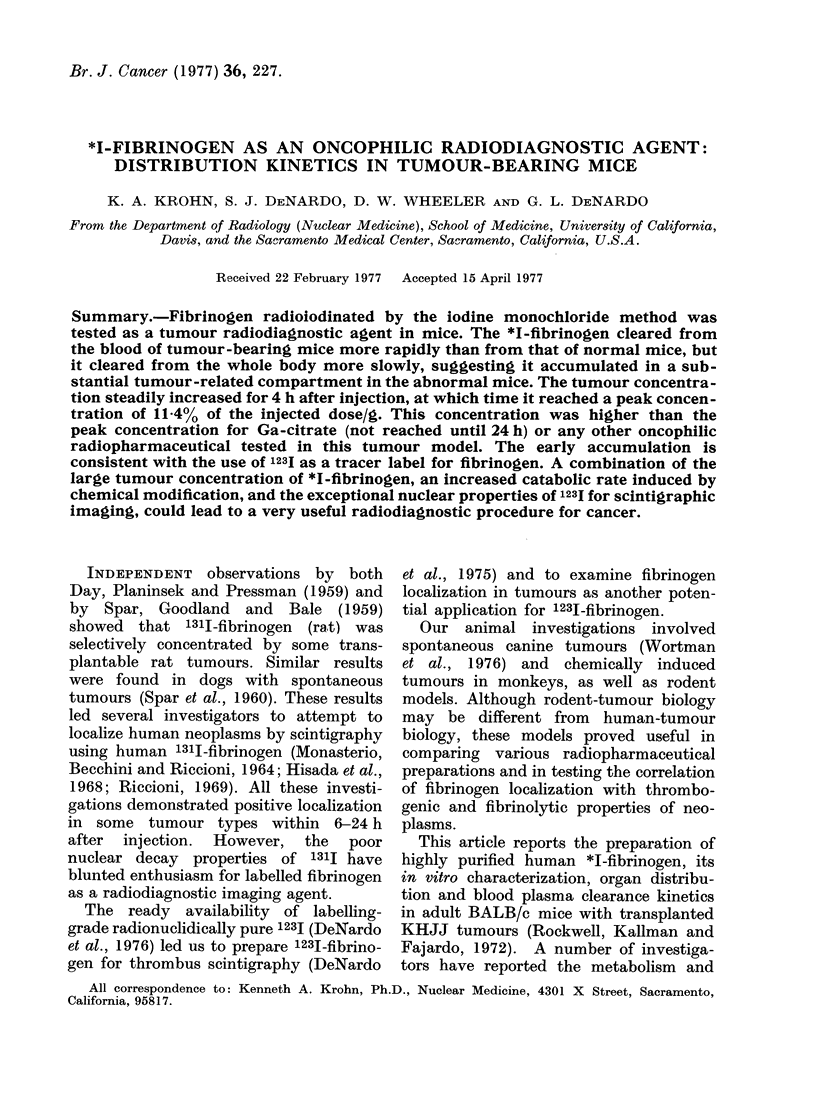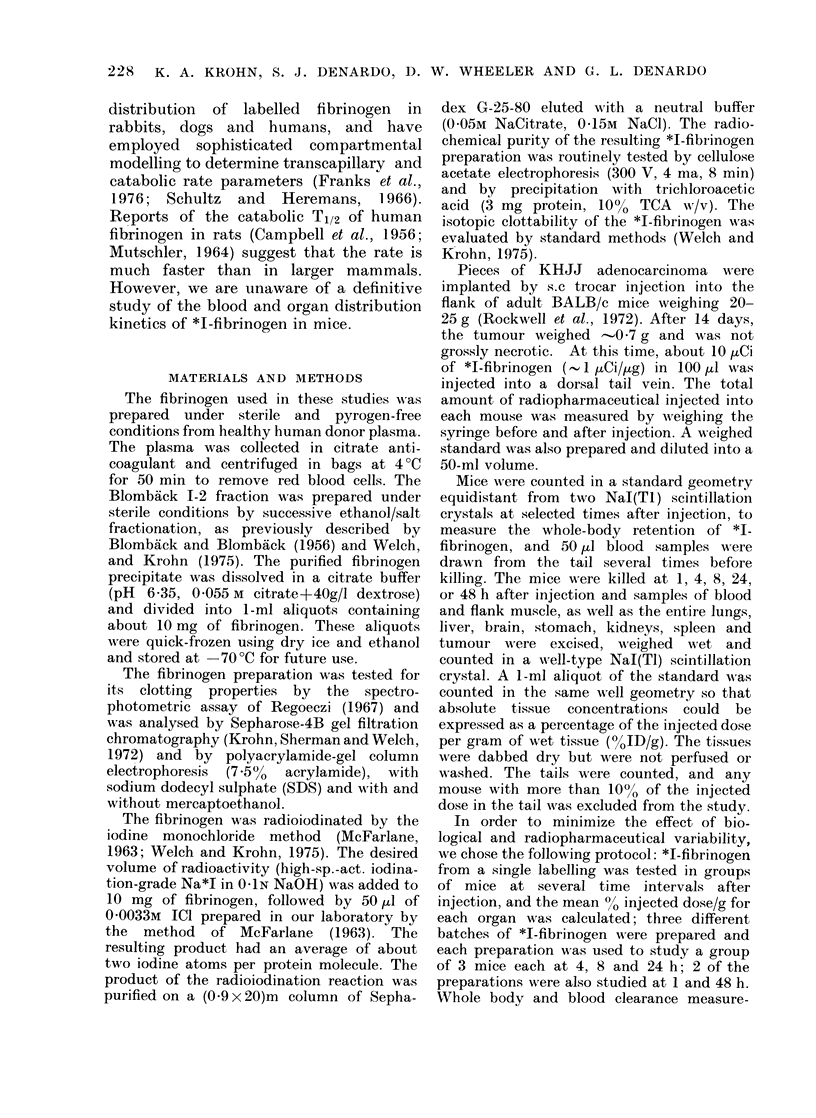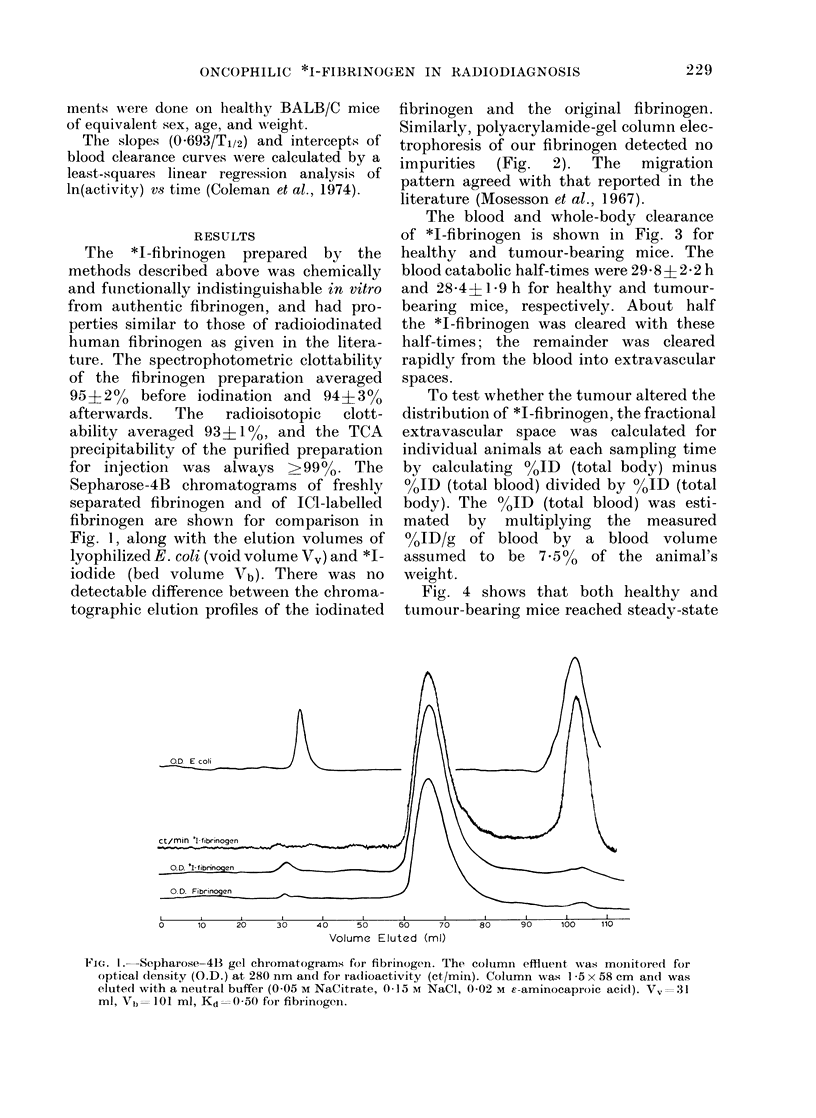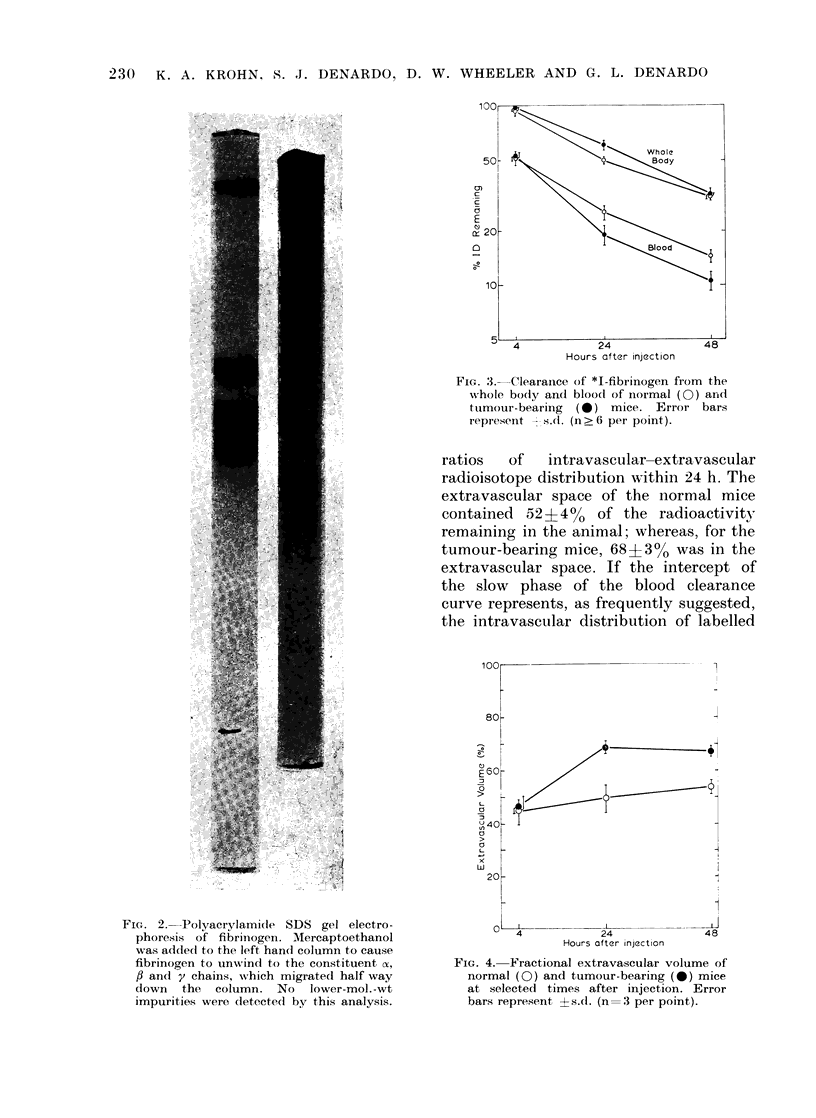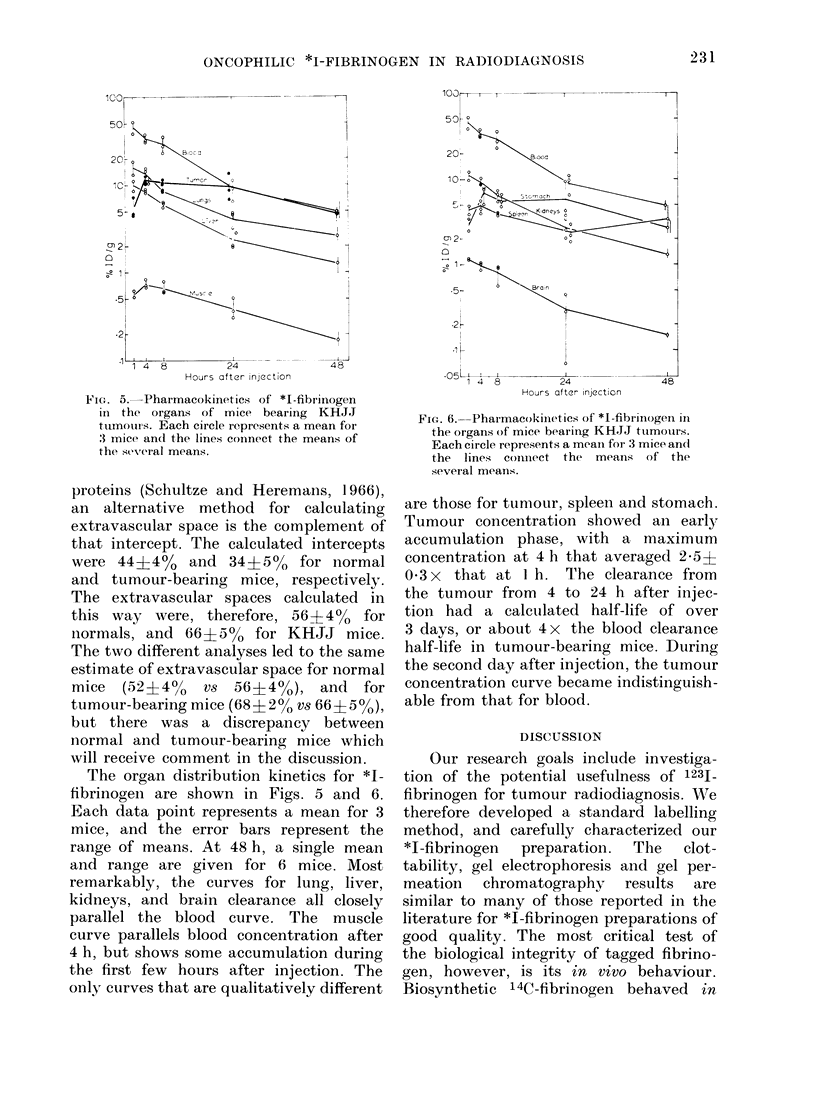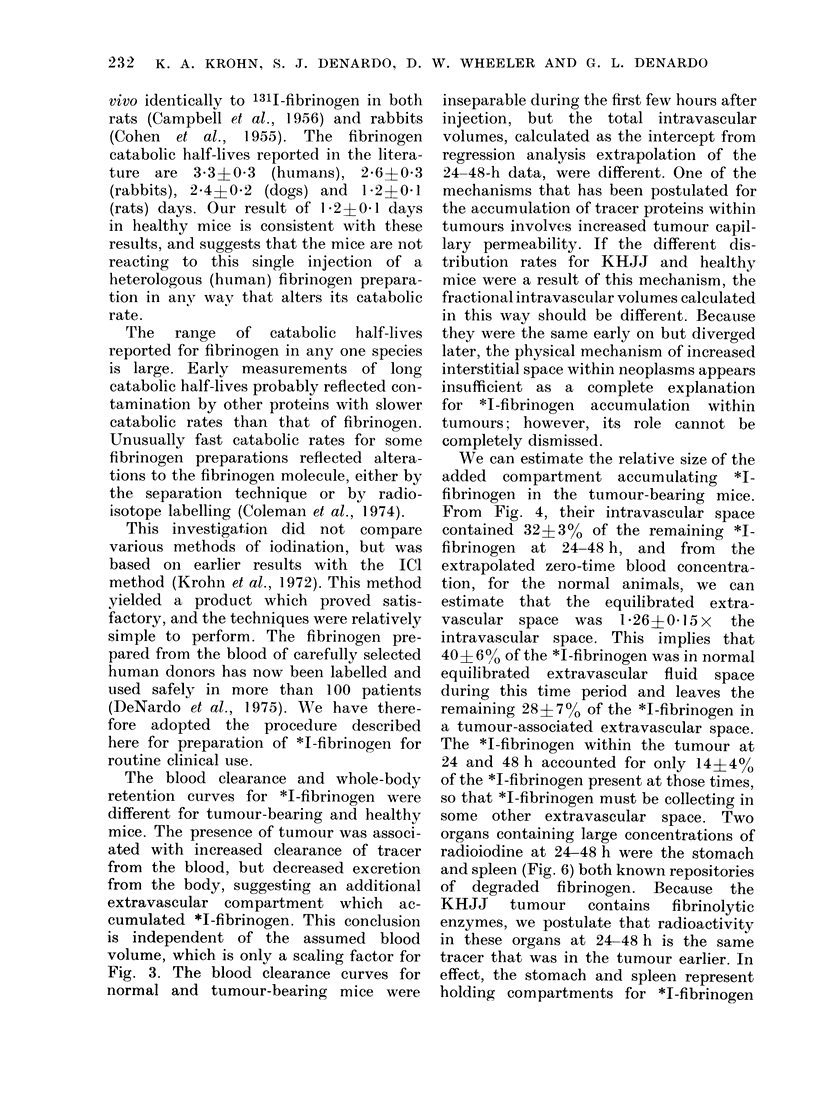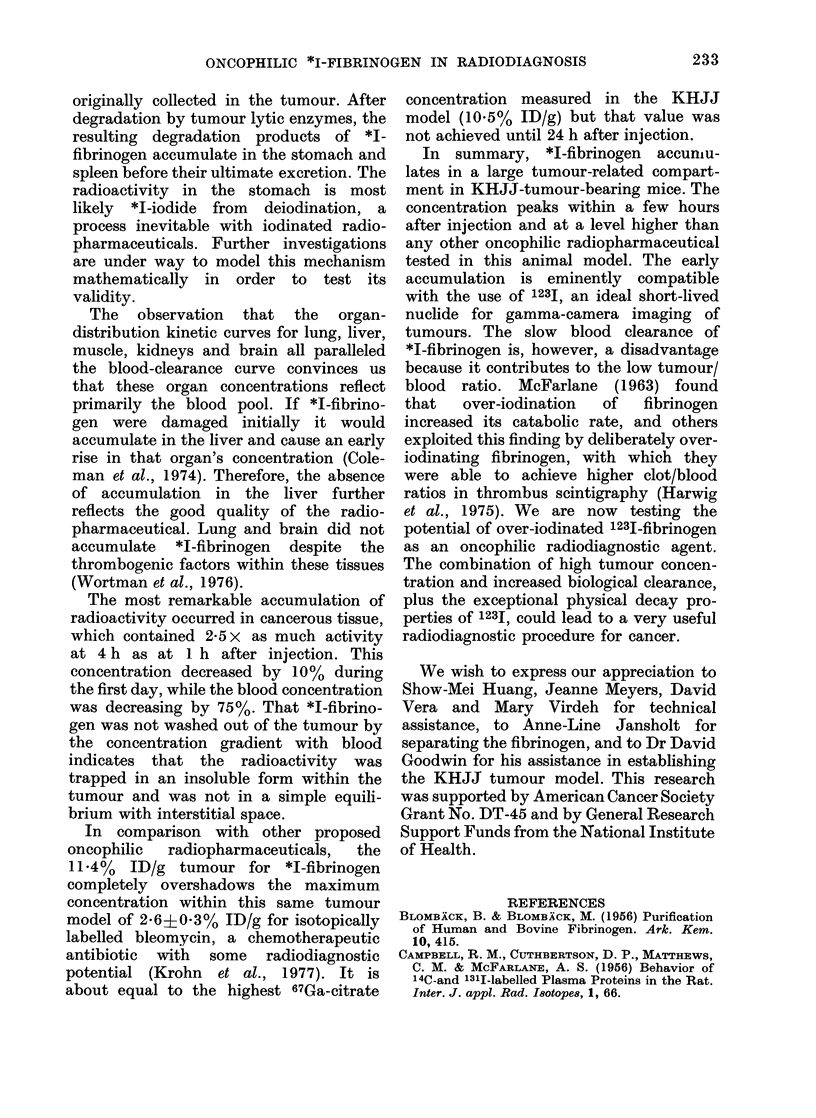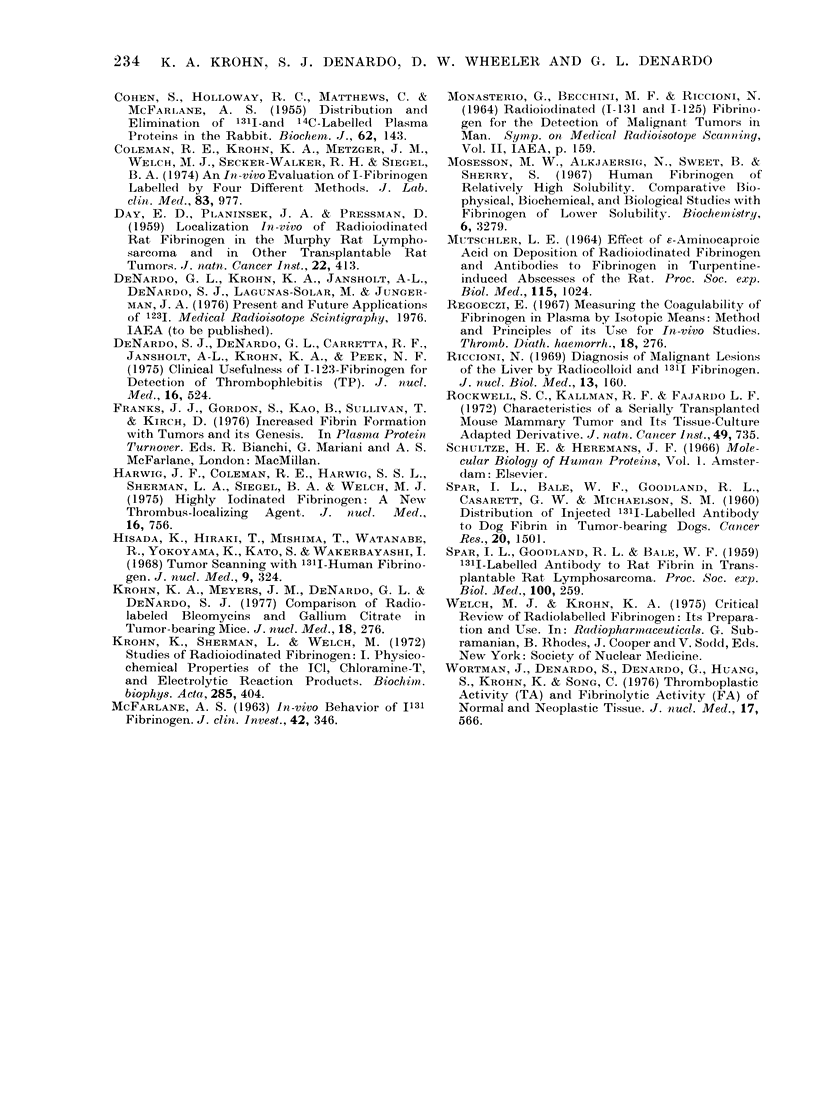# I-fibrinogen as an oncophilic radiodiagnostic agent: distribution kinetics in tumour-bearing mice.

**DOI:** 10.1038/bjc.1977.182

**Published:** 1977-08

**Authors:** K. A. Krohn, S. J. DeNardo, D. W. Wheeler, G. L. DeNardo

## Abstract

**Images:**


*Br. J. Cancer* (1977) **36**, 227.

### *I-FIBRINOGEN AS AN ONCOPHILIC RADIODIAGNOSTIC AGENT: DISTRIBUTION KINETICS IN TUMOUR-BEARING MICE

K. A. KROHN, S. J. DENARDO, D. W. WHEELER AND G. L. DENARDO

*From the Department of Radiology (Nuclear Medicine), School of Medicine, University of California, Davis, and the Sacramento Medical Center, Sacramento, California, U.S.A.*

Received 22 February 1977 Accepted 15 April 1977

**Summary.—Fibrinogen radioiodinated by the iodine monochloride method was tested as a tumour radiodiagnostic agent in mice. The *I-fibrinogen cleared from the blood of tumour-bearing mice more rapidly than from that of normal mice, but it cleared from the whole body more slowly, suggesting it accumulated in a substantial tumour-related compartment in the abnormal mice. The tumour concentration steadily increased for 4 h after injection, at which time it reached a peak concentration of 11·4% of the injected dose/g. This concentration was higher than the peak concentration for Ga-citrate (not reached until 24 h) or any other oncophilic radiopharmaceutical tested in this tumour model. The early accumulation is consistent with the use of $^{123}$I as a tracer label for fibrinogen. A combination of the large tumour concentration of *I-fibrinogen, an increased catabolic rate induced by chemical modification, and the exceptional nuclear properties of $^{123}$I for scintigraphic imaging, could lead to a very useful radiodiagnostic procedure for cancer.**

INDEPENDENT observations by both Day, Planinsek and Pressman (1959) and by Spar, Goodland and Bale (1959) showed that $^{131}$I-fibrinogen (rat) was selectively concentrated by some transplantable rat tumours. Similar results were found in dogs with spontaneous tumours (Spar *et al.*, 1960). These results led several investigators to attempt to localize human neoplasms by scintigraphy using human $^{131}$I-fibrinogen (Monasterio, Becchini and Riccioni, 1964; Hisada *et al.*, 1968; Riccioni, 1969). All these investigations demonstrated positive localization in some tumour types within 6–24 h after injection. However, the poor nuclear decay properties of $^{131}$I have blunted enthusiasm for labelled fibrinogen as a radiodiagnostic imaging agent.

The ready availability of labelling-grade radionuclidically pure $^{123}$I (DeNardo *et al.*, 1976) led us to prepare $^{123}$I-fibrinogen for thrombus scintigraphy (DeNardo *et al.*, 1975) and to examine fibrinogen localization in tumours as another potential application for $^{123}$I-fibrinogen.

Our animal investigations involved spontaneous canine tumours (Wortman *et al.*, 1976) and chemically induced tumours in monkeys, as well as rodent models. Although rodent-tumour biology may be different from human-tumour biology, these models proved useful in comparing various radiopharmaceutical preparations and in testing the correlation of fibrinogen localization with thrombogenic and fibrinolytic properties of neoplasms.

This article reports the preparation of highly purified human *I-fibrinogen, its *in vitro* characterization, organ distribution and blood plasma clearance kinetics in adult BALB/c mice with transplanted KHJJ tumours (Rockwell, Kallman and Fajardo, 1972). A number of investigators have reported the metabolism and

All correspondence to: Kenneth A. Krohn, Ph.D., Nuclear Medicine, 4301 X Street, Sacramento, California, 95817.

distribution of labelled fibrinogen in rabbits, dogs and humans, and have employed sophisticated compartmental modelling to determine transcapillary and catabolic rate parameters (Franks *et al.*, 1976; Schultz and Heremans, 1966). Reports of the catabolic $T_{1/2}$ of human fibrinogen in rats (Campbell *et al.*, 1956; Mutschler, 1964) suggest that the rate is much faster than in larger mammals. However, we are unaware of a definitive study of the blood and organ distribution kinetics of *I-fibrinogen in mice.

### MATERIALS AND METHODS

The fibrinogen used in these studies was prepared under sterile and pyrogen-free conditions from healthy human donor plasma. The plasma was collected in citrate anticoagulant and centrifuged in bags at 4 °C for 50 min to remove red blood cells. The Blombäck I-2 fraction was prepared under sterile conditions by successive ethanol/salt fractionation, as previously described by Blombäck and Blombäck (1956) and Welch, and Krohn (1975). The purified fibrinogen precipitate was dissolved in a citrate buffer (pH 6·35, 0·055 M citrate+40g/l dextrose) and divided into 1-ml aliquots containing about 10 mg of fibrinogen. These aliquots were quick-frozen using dry ice and ethanol and stored at −70 °C for future use.

The fibrinogen preparation was tested for its clotting properties by the spectrophotometric assay of Regoeczi (1967) and was analysed by Sepharose-4B gel filtration chromatography (Krohn, Sherman and Welch, 1972) and by polyacrylamide-gel column electrophoresis (7·5% acrylamide), with sodium dodecyl sulphate (SDS) and with and without mercaptoethanol.

The fibrinogen was radioiodinated by the iodine monochloride method (McFarlane, 1963; Welch and Krohn, 1975). The desired volume of radioactivity (high-sp.-act. iodination-grade Na*I in 0·1N NaOH) was added to 10 mg of fibrinogen, followed by 50 μl of 0·0033M ICl prepared in our laboratory by the method of McFarlane (1963). The resulting product had an average of about two iodine atoms per protein molecule. The product of the radioiodination reaction was purified on a (0·9×20)m column of Sephadex G-25-80 eluted with a neutral buffer (0·05M NaCitrate, 0·15M NaCl). The radiochemical purity of the resulting *I-fibrinogen preparation was routinely tested by cellulose acetate electrophoresis (300 V, 4 ma, 8 min) and by precipitation with trichloroacetic acid (3 mg protein, 10% TCA w/v). The isotopic clottability of the *I-fibrinogen was evaluated by standard methods (Welch and Krohn, 1975).

Pieces of KHJJ adenocarcinoma were implanted by s.c trocar injection into the flank of adult BALB/c mice weighing 20–25 g (Rockwell *et al.*, 1972). After 14 days, the tumour weighed ~0·7 g and was not grossly necrotic. At this time, about 10 μCi of *I-fibrinogen (~1 μCi/μg) in 100 μl was injected into a dorsal tail vein. The total amount of radiopharmaceutical injected into each mouse was measured by weighing the syringe before and after injection. A weighed standard was also prepared and diluted into a 50-ml volume.

Mice were counted in a standard geometry equidistant from two NaI(Tl) scintillation crystals at selected times after injection, to measure the whole-body retention of *I-fibrinogen, and 50 μl blood samples were drawn from the tail several times before killing. The mice were killed at 1, 4, 8, 24, or 48 h after injection and samples of blood and flank muscle, as well as the entire lungs, liver, brain, stomach, kidneys, spleen and tumour were excised, weighed wet and counted in a well-type NaI(Tl) scintillation crystal. A 1-ml aliquot of the standard was counted in the same well geometry so that absolute tissue concentrations could be expressed as a percentage of the injected dose per gram of wet tissue (%ID/g). The tissues were dabbed dry but were not perfused or washed. The tails were counted, and any mouse with more than 10% of the injected dose in the tail was excluded from the study.

In order to minimize the effect of biological and radiopharmaceutical variability, we chose the following protocol: *I-fibrinogen from a single labelling was tested in groups of mice at several time intervals after injection, and the mean % injected dose/g for each organ was calculated; three different batches of *I-fibrinogen were prepared and each preparation was used to study a group of 3 mice each at 4, 8 and 24 h; 2 of the preparations were also studied at 1 and 48 h. Whole body and blood clearance measurements were done on healthy BALB/C mice of equivalent sex, age, and weight.

The slopes ($0{\cdot}693/T_{1/2}$) and intercepts of blood clearance curves were calculated by a least-squares linear regression analysis of ln(activity) *vs* time (Coleman *et al.*, 1974).

### RESULTS

The *I-fibrinogen prepared by the methods described above was chemically and functionally indistinguishable *in vitro* from authentic fibrinogen, and had properties similar to those of radioiodinated human fibrinogen as given in the literature. The spectrophotometric clottability of the fibrinogen preparation averaged $95 \pm 2\%$ before iodination and $94 \pm 3\%$ afterwards. The radioisotopic clottability averaged $93 \pm 1\%$, and the TCA precipitability of the purified preparation for injection was always $\geq 99\%$. The Sepharose-4B chromatograms of freshly separated fibrinogen and of ICl-labelled fibrinogen are shown for comparison in Fig. 1, along with the elution volumes of lyophilized *E. coli* (void volume $V_v$) and *I-iodide (bed volume $V_b$). There was no detectable difference between the chromatographic elution profiles of the iodinated fibrinogen and the original fibrinogen. Similarly, polyacrylamide-gel column electrophoresis of our fibrinogen detected no impurities (Fig. 2). The migration pattern agreed with that reported in the literature (Mosesson *et al.*, 1967).

The blood and whole-body clearance of *I-fibrinogen is shown in Fig. 3 for healthy and tumour-bearing mice. The blood catabolic half-times were $29{\cdot}8 \pm 2{\cdot}2$ h and $28{\cdot}4 \pm 1{\cdot}9$ h for healthy and tumour-bearing mice, respectively. About half the *I-fibrinogen was cleared with these half-times; the remainder was cleared rapidly from the blood into extravascular spaces.

To test whether the tumour altered the distribution of *I-fibrinogen, the fractional extravascular space was calculated for individual animals at each sampling time by calculating %ID (total body) minus %ID (total blood) divided by %ID (total body). The %ID (total blood) was estimated by multiplying the measured %ID/g of blood by a blood volume assumed to be $7{\cdot}5\%$ of the animal's weight.

Fig. 4 shows that both healthy and tumour-bearing mice reached steady-state

FIG. 1.—Sepharose-4B gel chromatograms for fibrinogen. The column effluent was monitored for optical density (O.D.) at 280 nm and for radioactivity (ct/min). Column was $1{\cdot}5 \times 58$ cm and was eluted with a neutral buffer ($0{\cdot}05$ M NaCitrate, $0{\cdot}15$ M NaCl, $0{\cdot}02$ M $\varepsilon$-aminocaproic acid). $V_v = 31$ ml, $V_b = 101$ ml, $K_d = 0{\cdot}50$ for fibrinogen.

Fig. 2.—Polyacrylamide SDS gel electrophoresis of fibrinogen. Mercaptoethanol was added to the left hand column to cause fibrinogen to unwind to the constituent α, β and γ chains, which migrated half way down the column. No lower-mol.-wt impurities were detected by this analysis.

Fig. 3.—Clearance of *I-fibrinogen from the whole body and blood of normal (○) and tumour-bearing (●) mice. Error bars represent ±s.d. (n ≥ 6 per point).

ratios of intravascular–extravascular radioisotope distribution within 24 h. The extravascular space of the normal mice contained 52±4% of the radioactivity remaining in the animal; whereas, for the tumour-bearing mice, 68±3% was in the extravascular space. If the intercept of the slow phase of the blood clearance curve represents, as frequently suggested, the intravascular distribution of labelled

Fig. 4.—Fractional extravascular volume of normal (○) and tumour-bearing (●) mice at selected times after injection. Error bars represent ±s.d. (n = 3 per point).

FIG. 5.—Pharmacokinetics of *I-fibrinogen in the organs of mice bearing KHJJ tumours. Each circle represents a mean for 3 mice and the lines connect the means of the several means.

FIG. 6.—Pharmacokinetics of *I-fibrinogen in the organs of mice bearing KHJJ tumours. Each circle represents a mean for 3 mice and the lines connect the means of the several means.

proteins (Schultze and Heremans, 1966), an alternative method for calculating extravascular space is the complement of that intercept. The calculated intercepts were $44 \pm 4\%$ and $34 \pm 5\%$ for normal and tumour-bearing mice, respectively. The extravascular spaces calculated in this way were, therefore, $56 \pm 4\%$ for normals, and $66 \pm 5\%$ for KHJJ mice. The two different analyses led to the same estimate of extravascular space for normal mice ($52 \pm 4\%$ *vs* $56 \pm 4\%$), and for tumour-bearing mice ($68 \pm 2\%$ *vs* $66 \pm 5\%$), but there was a discrepancy between normal and tumour-bearing mice which will receive comment in the discussion.

The organ distribution kinetics for *I-fibrinogen are shown in Figs. 5 and 6. Each data point represents a mean for 3 mice, and the error bars represent the range of means. At 48 h, a single mean and range are given for 6 mice. Most remarkably, the curves for lung, liver, kidneys, and brain clearance all closely parallel the blood curve. The muscle curve parallels blood concentration after 4 h, but shows some accumulation during the first few hours after injection. The only curves that are qualitatively different are those for tumour, spleen and stomach. Tumour concentration showed an early accumulation phase, with a maximum concentration at 4 h that averaged $2{\cdot}5 \pm 0{\cdot}3 \times$ that at 1 h. The clearance from the tumour from 4 to 24 h after injection had a calculated half-life of over 3 days, or about $4 \times$ the blood clearance half-life in tumour-bearing mice. During the second day after injection, the tumour concentration curve became indistinguishable from that for blood.

### DISCUSSION

Our research goals include investigation of the potential usefulness of $^{123}$I-fibrinogen for tumour radiodiagnosis. We therefore developed a standard labelling method, and carefully characterized our *I-fibrinogen preparation. The clottability, gel electrophoresis and gel permeation chromatography results are similar to many of those reported in the literature for *I-fibrinogen preparations of good quality. The most critical test of the biological integrity of tagged fibrinogen, however, is its *in vivo* behaviour. Biosynthetic $^{14}$C-fibrinogen behaved *in*

*vivo* identically to $^{131}$I-fibrinogen in both rats (Campbell *et al.*, 1956) and rabbits (Cohen *et al.*, 1955). The fibrinogen catabolic half-lives reported in the literature are $3{\cdot}3\pm0{\cdot}3$ (humans), $2{\cdot}6\pm0{\cdot}3$ (rabbits), $2{\cdot}4\pm0{\cdot}2$ (dogs) and $1{\cdot}2\pm0{\cdot}1$ (rats) days. Our result of $1{\cdot}2\pm0{\cdot}1$ days in healthy mice is consistent with these results, and suggests that the mice are not reacting to this single injection of a heterologous (human) fibrinogen preparation in any way that alters its catabolic rate.

The range of catabolic half-lives reported for fibrinogen in any one species is large. Early measurements of long catabolic half-lives probably reflected contamination by other proteins with slower catabolic rates than that of fibrinogen. Unusually fast catabolic rates for some fibrinogen preparations reflected alterations to the fibrinogen molecule, either by the separation technique or by radioisotope labelling (Coleman *et al.*, 1974).

This investigation did not compare various methods of iodination, but was based on earlier results with the ICl method (Krohn *et al.*, 1972). This method yielded a product which proved satisfactory, and the techniques were relatively simple to perform. The fibrinogen prepared from the blood of carefully selected human donors has now been labelled and used safely in more than 100 patients (DeNardo *et al.*, 1975). We have therefore adopted the procedure described here for preparation of *I-fibrinogen for routine clinical use.

The blood clearance and whole-body retention curves for *I-fibrinogen were different for tumour-bearing and healthy mice. The presence of tumour was associated with increased clearance of tracer from the blood, but decreased excretion from the body, suggesting an additional extravascular compartment which accumulated *I-fibrinogen. This conclusion is independent of the assumed blood volume, which is only a scaling factor for Fig. 3. The blood clearance curves for normal and tumour-bearing mice were inseparable during the first few hours after injection, but the total intravascular volumes, calculated as the intercept from regression analysis extrapolation of the 24–48-h data, were different. One of the mechanisms that has been postulated for the accumulation of tracer proteins within tumours involves increased tumour capillary permeability. If the different distribution rates for KHJJ and healthy mice were a result of this mechanism, the fractional intravascular volumes calculated in this way should be different. Because they were the same early on but diverged later, the physical mechanism of increased interstitial space within neoplasms appears insufficient as a complete explanation for *I-fibrinogen accumulation within tumours; however, its role cannot be completely dismissed.

We can estimate the relative size of the added compartment accumulating *I-fibrinogen in the tumour-bearing mice. From Fig. 4, their intravascular space contained $32\pm3\%$ of the remaining *I-fibrinogen at 24–48 h, and from the extrapolated zero-time blood concentration, for the normal animals, we can estimate that the equilibrated extravascular space was $1{\cdot}26\pm0{\cdot}15\times$ the intravascular space. This implies that $40\pm6\%$ of the *I-fibrinogen was in normal equilibrated extravascular fluid space during this time period and leaves the remaining $28\pm7\%$ of the *I-fibrinogen in a tumour-associated extravascular space. The *I-fibrinogen within the tumour at 24 and 48 h accounted for only $14\pm4\%$ of the *I-fibrinogen present at those times, so that *I-fibrinogen must be collecting in some other extravascular space. Two organs containing large concentrations of radioiodine at 24–48 h were the stomach and spleen (Fig. 6) both known repositories of degraded fibrinogen. Because the KHJJ tumour contains fibrinolytic enzymes, we postulate that radioactivity in these organs at 24–48 h is the same tracer that was in the tumour earlier. In effect, the stomach and spleen represent holding compartments for *I-fibrinogen originally collected in the tumour. After degradation by tumour lytic enzymes, the resulting degradation products of *I-fibrinogen accumulate in the stomach and spleen before their ultimate excretion. The radioactivity in the stomach is most likely *I-iodide from deiodination, a process inevitable with iodinated radiopharmaceuticals. Further investigations are under way to model this mechanism mathematically in order to test its validity.

The observation that the organ-distribution kinetic curves for lung, liver, muscle, kidneys and brain all paralleled the blood-clearance curve convinces us that these organ concentrations reflect primarily the blood pool. If *I-fibrinogen were damaged initially it would accumulate in the liver and cause an early rise in that organ's concentration (Coleman *et al.*, 1974). Therefore, the absence of accumulation in the liver further reflects the good quality of the radiopharmaceutical. Lung and brain did not accumulate *I-fibrinogen despite the thrombogenic factors within these tissues (Wortman *et al.*, 1976).

The most remarkable accumulation of radioactivity occurred in cancerous tissue, which contained 2·5× as much activity at 4 h as at 1 h after injection. This concentration decreased by 10% during the first day, while the blood concentration was decreasing by 75%. That *I-fibrinogen was not washed out of the tumour by the concentration gradient with blood indicates that the radioactivity was trapped in an insoluble form within the tumour and was not in a simple equilibrium with interstitial space.

In comparison with other proposed oncophilic radiopharmaceuticals, the 11·4% ID/g tumour for *I-fibrinogen completely overshadows the maximum concentration within this same tumour model of $2{\cdot}6 \pm 0{\cdot}3\%$ ID/g for isotopically labelled bleomycin, a chemotherapeutic antibiotic with some radiodiagnostic potential (Krohn *et al.*, 1977). It is about equal to the highest $^{67}$Ga-citrate concentration measured in the KHJJ model (10·5% ID/g) but that value was not achieved until 24 h after injection.

In summary, *I-fibrinogen accumulates in a large tumour-related compartment in KHJJ-tumour-bearing mice. The concentration peaks within a few hours after injection and at a level higher than any other oncophilic radiopharmaceutical tested in this animal model. The early accumulation is eminently compatible with the use of $^{123}$I, an ideal short-lived nuclide for gamma-camera imaging of tumours. The slow blood clearance of *I-fibrinogen is, however, a disadvantage because it contributes to the low tumour/blood ratio. McFarlane (1963) found that over-iodination of fibrinogen increased its catabolic rate, and others exploited this finding by deliberately over-iodinating fibrinogen, with which they were able to achieve higher clot/blood ratios in thrombus scintigraphy (Harwig *et al.*, 1975). We are now testing the potential of over-iodinated $^{123}$I-fibrinogen as an oncophilic radiodiagnostic agent. The combination of high tumour concentration and increased biological clearance, plus the exceptional physical decay properties of $^{123}$I, could lead to a very useful radiodiagnostic procedure for cancer.

We wish to express our appreciation to Show-Mei Huang, Jeanne Meyers, David Vera and Mary Virdeh for technical assistance, to Anne-Line Jansholt for separating the fibrinogen, and to Dr David Goodwin for his assistance in establishing the KHJJ tumour model. This research was supported by American Cancer Society Grant No. DT-45 and by General Research Support Funds from the National Institute of Health.

### REFERENCES

BLOMBÄCK, B. & BLOMBÄCK, M. (1956) Purification of Human and Bovine Fibrinogen. *Ark. Kem.* **10**, 415.

CAMPBELL, R. M., CUTHBERTSON, D. P., MATTHEWS, C. M. & MCFARLANE, A. S. (1956) Behavior of $^{14}$C-and $^{131}$I-labelled Plasma Proteins in the Rat. *Inter. J. appl. Rad. Isotopes*, **1**, 66.

COHEN, S., HOLLOWAY, R. C., MATTHEWS, C. & McFARLANE, A. S. (1955) Distribution and Elimination of $^{131}$I-and $^{14}$C-Labelled Plasma Proteins in the Rabbit. *Biochem. J.*, **62**, 143.

COLEMAN, R. E., KROHN, K. A., METZGER, J. M., WELCH, M. J., SECKER-WALKER, R. H. & SIEGEL, B. A. (1974) An *In-vivo* Evaluation of I-Fibrinogen Labelled by Four Different Methods. *J. Lab. clin. Med.*, **83**, 977.

DAY, E. D., PLANINSEK, J. A. & PRESSMAN, D. (1959) Localization *In-vivo* of Radioiodinated Rat Fibrinogen in the Murphy Rat Lymphosarcoma and in Other Transplantable Rat Tumors. *J. natn. Cancer Inst.*, **22**, 413.

DENARDO, G. L., KROHN, K. A., JANSHOLT, A-L., DENARDO, S. J., LAGUNAS-SOLAR, M. & JUNGERMAN, J. A. (1976) Present and Future Applications of $^{123}$I. *Medical Radioisotope Scintigraphy*, 1976. IAEA (to be published).

DENARDO, S. J., DENARDO, G. L., CARRETTA, R. F., JANSHOLT, A-L., KROHN, K. A., & PEEK, N. F. (1975) Clinical Usefulness of I-123-Fibrinogen for Detection of Thrombophlebitis (TP). *J. nucl. Med.*, **16**, 524.

FRANKS, J. J., GORDON, S., KAO, B., SULLIVAN, T. & KIRCH, D. (1976) Increased Fibrin Formation with Tumors and its Genesis. In *Plasma Protein Turnover*. Eds. R. Bianchi, G. Mariani and A. S. McFarlane, London: MacMillan.

HARWIG, J. F., COLEMAN, R. E., HARWIG, S. S. L., SHERMAN, L. A., SIEGEL, B. A. & WELCH, M. J. (1975) Highly Iodinated Fibrinogen: A New Thrombus-localizing Agent. *J. nucl. Med.*, **16**, 756.

HISADA, K., HIRAKI, T., MISHIMA, T., WATANABE, R., YOKOYAMA, K., KATO, S. & WAKERBAYASHI, I. (1968) Tumor Scanning with $^{131}$I-Human Fibrinogen. *J. nucl. Med.*, **9**, 324.

KROHN, K. A., MEYERS, J. M., DENARDO, G. L. & DENARDO, S. J. (1977) Comparison of Radiolabeled Bleomycins and Gallium Citrate in Tumor-bearing Mice. *J. nucl. Med.*, **18**, 276.

KROHN, K., SHERMAN, L. & WELCH, M. (1972) Studies of Radioiodinated Fibrinogen: I. Physicochemical Properties of the ICl, Chloramine-T, and Electrolytic Reaction Products. *Biochim. biophys. Acta*, **285**, 404.

McFARLANE, A. S. (1963) *In-vivo* Behavior of $I^{131}$ Fibrinogen. *J. clin. Invest.*, **42**, 346.

MONASTERIO, G., BECCHINI, M. F. & RICCIONI, N. (1964) Radioiodinated (I-131 and I-125) Fibrinogen for the Detection of Malignant Tumors in Man. *Symp. on Medical Radioisotope Scanning*, Vol. II, IAEA, p. 159.

MOSESSON, M. W., ALKJAERSIG, N., SWEET, B. & SHERRY, S. (1967) Human Fibrinogen of Relatively High Solubility. Comparative Biophysical, Biochemical, and Biological Studies with Fibrinogen of Lower Solubility. *Biochemistry*, **6**, 3279.

MUTSCHLER, L. E. (1964) Effect of ε-Aminocaproic Acid on Deposition of Radioiodinated Fibrinogen and Antibodies to Fibrinogen in Turpentine-induced Abscesses of the Rat. *Proc. Soc. exp. Biol. Med.*, **115**, 1024.

REGOECZI, E. (1967) Measuring the Coagulability of Fibrinogen in Plasma by Isotopic Means: Method and Principles of its Use for *In-vivo* Studies. *Thromb. Diath. haemorrh.*, **18**, 276.

RICCIONI, N. (1969) Diagnosis of Malignant Lesions of the Liver by Radiocolloid and $^{131}$I Fibrinogen. *J. nucl. Biol. Med.*, **13**, 160.

ROCKWELL, S. C., KALLMAN, R. F. & FAJARDO L. F. (1972) Characteristics of a Serially Transplanted Mouse Mammary Tumor and Its Tissue-Culture Adapted Derivative. *J. natn. Cancer Inst.*, **49**, 735.

SCHULTZE, H. E. & HEREMANS, J. F. (1966) *Molecular Biology of Human Proteins*, Vol. 1. Amsterdam: Elsevier.

SPAR, I. L., BALE, W. F., GOODLAND, R. L., CASARETT, G. W. & MICHAELSON, S. M. (1960) Distribution of Injected $^{131}$I-Labelled Antibody to Dog Fibrin in Tumor-bearing Dogs. *Cancer Res.*, **20**, 1501.

SPAR, I. L., GOODLAND, R. L. & BALE, W. F. (1959) $^{131}$I-Labelled Antibody to Rat Fibrin in Transplantable Rat Lymphosarcoma. *Proc. Soc. exp. Biol. Med.*, **100**, 259.

WELCH, M. J. & KROHN, K. A. (1975) Critical Review of Radiolabelled Fibrinogen: Its Preparation and Use. In: *Radiopharmaceuticals*. G. Subramanian, B. Rhodes, J. Cooper and V. Sodd, Eds. New York: Society of Nuclear Medicine.

WORTMAN, J., DENARDO, S., DENARDO, G., HUANG, S., KROHN, K. & SONG, C. (1976) Thromboplastic Activity (TA) and Fibrinolytic Activity (FA) of Normal and Neoplastic Tissue. *J. nucl. Med.*, **17**, 566.